# Fast and Simple Fabrication of Superhydrophobic Coating by Polymer Induced Phase Separation

**DOI:** 10.3390/nano9030411

**Published:** 2019-03-11

**Authors:** Yu-Ping Zhang, Pan-Pan Li, Peng-Fei Liu, Wan-Qing Zhang, Ji-Chao Wang, Cheng-Xing Cui, Xiang-Jun Li, Ling-Bo Qu

**Affiliations:** 1 Henan Institute of Science and Technology, Xinxiang 453000, China; ccxcyh@gmail.com (P.-P.L.); lpf8856@163.com (P.-F.L.); zhangwqzzu@163.com (W.-Q.Z.); wangjichao@hist.edu.cn (J.-C.W.); chengxingcui@hist.edu.cn (C.-X.C.); 2 College of Chemistry and Molecular Engineering, Zhengzhou University, Henan 450001, China; 3 School of Chemical Sciences, University of Chinese Academy of Sciences, Beijing 100049, China; lixiangj@ucas.ac.cn

**Keywords:** polymerization, superhydrophobic material, microwave irradiation, UV irradiation, nitrite

## Abstract

Polymerization-induced phase separation is comparatively applied to fabricating a superhydrophobic micro/nano surface by microwave and ultraviolet (UV) irradiation. The monolithic coatings can be tailored easily on different substrates with excellent chemical and mechanical stability in rigid conditions. Importantly, the patterned filter paper is successfully used for the colorimetric detection of nitrite handily.

## 1. Introduction

Wetting phenomena of solid surface are ubiquitous in nature as well as in many commercial applications including oil/water separation, membrane engineering and microfluidics [1,2,3,4,5]. Surface with a static water contact angle (WCAs) greater than 150° and roll-off angles (RAs) below 10° for water is generally defined as superhydrophobic one [6,7]. Numerous approaches have been attempted to fabricate superhydrophobic materials, which include chemical/mechanical etching [8], sol–gel process [9], layer-by-layer self-assembly [10], electrospinning [11], electrodeposition and CVD (chemical vapor deposition) [12,13]. Unfortunately, the preparation procedures are usually complicated and time-consuming, not to mention their dependence on multi-step procedures or some special instruments as well as post-modification by expensive chemicals with low surface energy. Especially, weak adhesion between the surfaces and the substrates usually impedes their practical application. Therefore, it is necessary to find more convenient methods and select suitable materials for fabricating superhydrophobic coatings [14,15,16]. Monolithic materials have been widely used as the absorbent in sample pretreatment and the stationary phase in HPLC, μ-HPLC and capillary electrochromatography (CEC) due to their unique advantages such as fast separation, high-linear flow velocities and nonrequirement of frits [17]. It is well known that the stationary phases in reverse phase liquid chromatography (RPLC) are common hydrophobic materials but very few literatures are reported how to modify the hydrophobic stationary phase to superhydrophobic one [18,19,20].

Levkin and co-workers prepared superhydrophobic coatings of poly (butyl methacrylate-co-ethylene dimethacrylate, BMA-EDMA) and poly(styrene-co-1,4-divinylbenzene, ST-DVD) but thermally initiated polymerization was carried out at a temperature of 70 °C for 24 h and a detailed investigation of ruggedness was not considered [19]. Afterwards, Kato and Sato reported a ultraviolet (UV) triggered phase-separation process to convert a coated precursor to superhydrophobic coatings but soluble linear polymers should be added to the porogenic agents for the process of polymerization-induced phase separation (PIPS) [20]. Afterwards, microporous poly(BMA-co-EDMA) surfaces were prepared via UV-initiated free-radical polymerization. Subsequent infusion of fluorocarbon lubricants into the porous microtexture resulted in liquid-repellent slippery surfaces with a marine antibiofouling property [21].

In the present communication, we focus on a more simple, rapid and versatile approach to modify hydrophobic polymer monoliths to the superhydrophobic one under an open-air environment. For the first time, PIPS is carried out through the optimal selection of monomer and porogen with the help of microwave and UV irradiation (see Scheme S1). The fabrication of superhydrophobic monoliths take only 4 min for microwave-curing method. The operation includes three steps: (1) Dropping the polymerization mixture on a substrate, (2) Microwave or UV irradiation to induce phase separation and (3) Ethanol flushing the porous monolithic surface. By optimizing the appropriate composition of the porogenic agents, the micro- and nanoscale superhydrophobic surface is logically tailored.

## 2. Materials and Methods

Butyl methacrylate (BMA), ethylene dimethacrylate (EDMA), methacrylic acid (MAA), 3-(methacryloyloxy)propyl]trimethoxysilane (γ-MPTMS), ethanol, isooctane, toluene, butanediol, lauryl alcohol, azobisisobutyronitrile (AIBN) used as a thermal initiator are purchased from Aladdin Chemical Reagent Co., Ltd. (Shanghai, China) and Tianjin Kermel Chemical Reagent Co., Ltd., (Tianjin, China). Irgacure 1800 as a UV photo-initiator is donated by Ciba-Geigy (Tianjing, China).

The glass slide is pretreated to create binding sites for the attachments of the polymeric materials. Firstly, the glass slide is immersed in 1M NaOH for 30 min and then with 0.1M HCI for 30 min. Subsequently, it is flushed with H_2_O for several times and then dried by passing nitrogen gas. The glass plates are immersing in a silanizing solution, a solution of 50% (*v*/*v*) of γ-MPTMS in ethanol overnight at 60 °C, then dried for the next use. The purpose of surface pretreatment is to increase the concentration of surface silanol groups. Since silanol groups on the glass surface represent the principal binding sites for in situ created polymer-based monolith, higher concentration of these binding sites on the glass surface would facilitate the coating formation of highly secured organic-based monolith [18,19,20]. Microwave irradiation is carried out in a simple home microwave oven with a common frequency of 2450 HZ. An XL-1500 UV cross-linker (Spectronics Corp., Westbury, NY, USA) is equipped with six 15 W blacklight tubes, in which the dropping surfaces of different substrates are irradiated with a total dose of 0.9 J/cm^2^ at a wavelength of 365 nm.

**Scheme 1:**
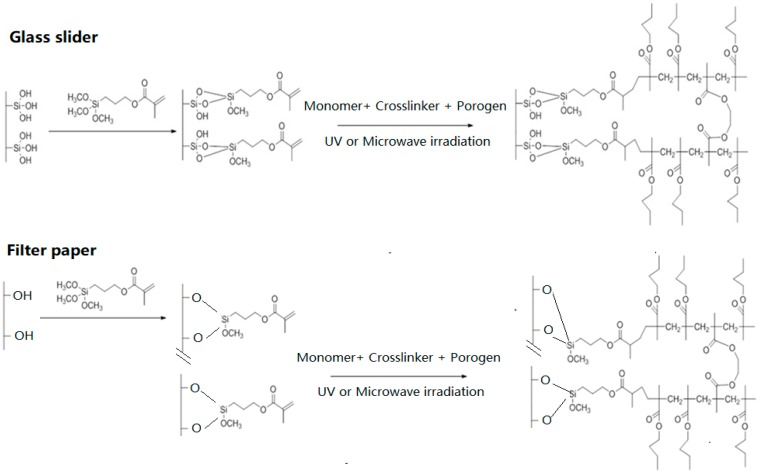
Illustration of the fabrication procedures and relative mechanism on the glass slide and filter paper.

Briefly, a polymerization solution consisting of monomer (BMA or MAA), crosslinker (EDMA), porogenic mixtures and a thermal initiator (AIBN) or a UV photo-initiator (Irgacure 1800) is spread evenly on a substrate such as the glass slide, filter paper, Cu foil, Al foil and PTFE film, then irradiated by microwave or UV. The amount of both initiators are about 1.0% (*w*/*v*) of the used monomers. In the PIPS process, the solvents are miscible with the cross-linkable monomers but immiscible with the generating network polymers, so that a phase-separated state can be obtained. After flushing off unreacted compositions, a monolithic surface with a defined wettability and ruggedness is formed. Illustration of the fabrication procedures and relative mechanism is shown in Scheme 1.

## 3. Results and Discussion

### 3.1. Preparation of the Monolithic Coatings by Microwave and UV Irradiations

Initial experiments with the help of UV irradiation are attempted to prepare the monolithic coating on the substrate of glass slides using the typical monomers of methacrylic acid (MAA) and butyl methacrylate (BMA), EDMA as the crosslinker, toluene and isooctane as the binary porogens. The polymerization formula for UV irradiation referred to our previous work [22,23]. Table S1 indicates that the final WCAs are changed in the range of 71–111° for the resulted monolithic surfaces in the absence and presence of porogens. The fact that no superhydrophobic surface is formed is probably attributed to the use of hydrophilic monomer of MAA. Further attempt is carried out under the given condition that the amount of BMA and EDMA used are fixed, selection of both solvents of butanediol and 1-propanol is found to benefit for the forming of hydrophobic surfaces under an open-air condition. From the results in Table 1, it shows that all fabricated monolithic materials of poly(BMA-co-EDMA) are hydrophobic with the WCAs changed in the range of 94°–109°.

In order to transform the hydrophobic monolith to superhydrophobic one, further optimization is needed using relatively low volatility, high viscosity and nonpolar porogens so that high quality of the phase separation can be achieved by microwave and UV irradiation [12]. Typically, a monomer mixture containing a 3:2 volume ratio of BMA and EDMA is fixed but the ratio of lauryl alcohol and 1,4-butanediol is changed for the optimization (See Table S2). In all experiments, the volume ratio (2/3) of monomer mixture and binary porogens is kept constant. Microwave and UV irradiation are carried out in 5 min and 30 min using polymeric mixtures 1–6, respectively, so that some coatings with superhydrophobicity are obtained on the glass slides. Table S2 summarizes the preparation conditions for the monolithic coatings and their WCAs using microwave and UV irradiation. For mixture 1 without porogenic agent, the formed non-porous polymers of poly(BMA-EDMA) for both microwave and UV irradiation are very hydrophilic. Fortunately, the porous monoliths for mixture 5 are obtained with the largest WCAs of 170° and 155°, respectively. After further chemical vapor deposition (CVD) using perfluoro-1,1,2,2-tetrahydrooctyltrichlorosilane (FOTS), the WCAs increase and all monolithic surfaces becoming superhydrophobic but mixtures 1 and 6 (see Figure S1). The very high contact angle (>150°) and extremely low roll-off angle (<5°) clearly indicate that all as-prepared porous surfaces fabricated by both approaches are superhydrophobic. It is quite difficult to dispense water droplets on the monolithic surfaces manually using pipettes. From the results, it is evident that the replacement of 1-propanol with lauryl alcohol leads to a well-developed phase separation state.

### 3.2. Characterization and Evaluation

**Figure 1:**
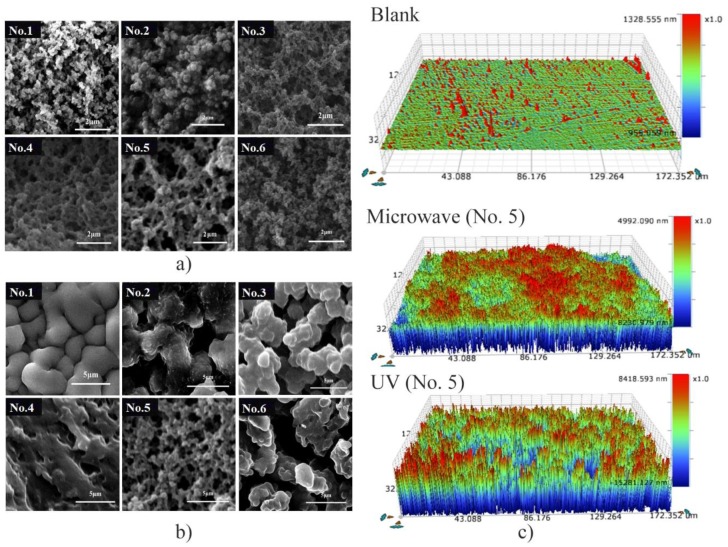
Left: Scanning electron microscopy (SEM) of polymeric coatings fabricated by microwave (**a**) and UV (**b**) irradiation; Right: the comparative roughness of the blank glass slide, the optimal microwave-cured coating, the optimal UV-cured coatings (**c**).

Surface morphology of the glass slides are obtained by using scanning electron microscopy (SEM) in Figure 1a,b. It is observed that very small spherical particles of nanometer size that are generated during phase-separation and agglomerate forming the interconnected network for two approaches. Interestingly, it is clear that the smallest particle size and the distribution of more regular pore size is obtained for mixture 5 irradiated by UV. These irregular agglomerates consisting of nanometer size polymers and their interconnected network give rise to the Cassie-Baxter state by increasing the effective roughness of the surfaces. Fourier transform-infrared spectroscopy (FT-IR) indicates that poly(BMA-co-EDMA) is successfully formed with some typical characteristic peaks (See Figure S2a). The adsorption peak located at 2961 cm^−1^ is attributed to the stretching/vibration of –CH_3_ and –CH_2_, while the (C=) stretching/vibration of acrylate occurred located at 1735 cm^−1^. The carbonyl (C=O) stretching band occurs at 1600 to 1850 cm^−1^. The band at 1458 cm^−1^ is attributed to the skeleton vibration peak of benzene ring. Thermogravimetric analysis (TG) indicates that there are 47% composition lost at a temperature of 240 °C and the rest lost at a temperature of 400 °C. The former is the unreacted monomer and porogens and the latter is the cracked polymers of poly(BMA-co-EDMA) (See Figure S2b).

The elemental analysis (i.e., chemical characterization) of the monoliths is carried out by using X-ray photoelectron spectroscopy energy (XPS). As shown in Figure S3, the results show that the coating are composed of carbon (weight % = 90.0, atomic % = 83.4), oxygen (weight % = 10, atomic % = 16.6) for microwave-cured surface; and carbon (weight % = 85.8, atomic % = 79.7), oxygen (weight % = 14.2, atomic % = 20.3) for UV-cured surface, respectively, as the main constituents with carbon having the highest percentage.

The surface topography and nanoscale asperities are measured using a 3D surface profilometer (see Figure 1c) for the optimal polymers. The roughness values obtained from the measurements are as follows: average roughness (Ra) = 16 nm, root mean squared roughness (Rq) = 28 nm for the blank glass slide; Ra = 903 nm, Rq = 1141 nm for the microwave-cured glass slide; and a comparative Ra = 2460 nm, Rq = 3103 nm for the UV-cured glass slide. Interestingly, thinner coating with a thickness of 11 μm for the microwave-cured glass slide led to a larger RAs than the UV-cured coating with 96 μm (see Figure S4).

Self-cleaning and water drop bouncing for the microwave-cured coating of mixture 5 were carried out. As shown in Figures S5 and S6 (Videos S1 and S2), the sand particles on the surface are readily removed by spraying with water and the water droplets can bounce up and down on the surface. Meanwhile, the porous polymers ground into particles are pressed on the gloves and water droplet can roll off easily from their faces, respectively (see Video S3).

**Figure 2:**
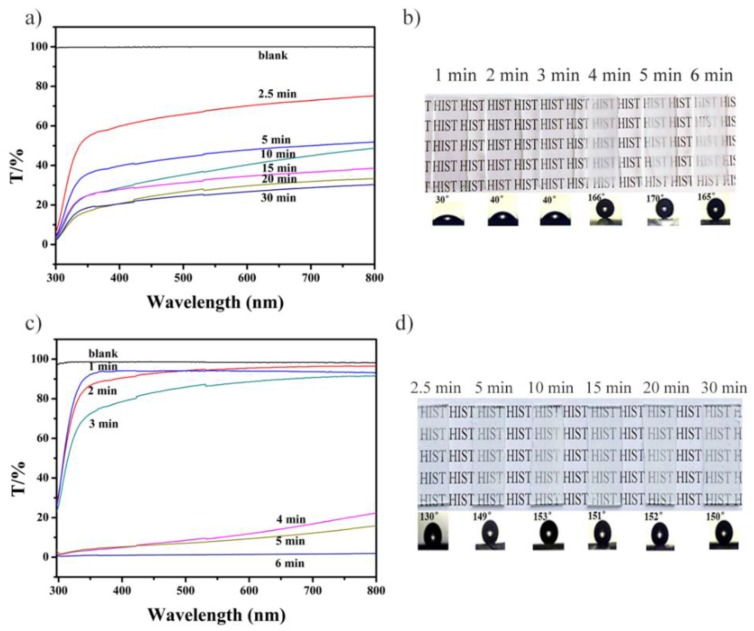
UV-Vis transmittance spectra (**a**), their relative transparency and their wetting property (**b**) for the microwave-irradiated coatings of mixture 5. UV-Vis transmittance spectra (**c**), their relative transparency and their wetting property (**d**) for the UV-irradiated coatings of mixture 5.

In order to attain a suitable compromise between high transparency and superhydrophobicity, mixture 5 with the largest WCAs for both methods is selected for the optimization of irradiation time. The results show the monolithic coatings are transformed to superhydrophobic only within 4 min for microwave irradiation but the transparency is hard to further improved (see Figure 2). In contrast, the monolithic coatings are transformed from hydrophobic (WCA = 125°) to superhydrophobic within 5–10 min for the UV-curing surface. UV-visible transmittance spectra indicate that longer irradiation time lead to less transparency due to the more monoliths formed. Superhydrophobicity is attained through a combination of suitable roughness and low surface energy material. Whilst transparency is generally achieved by reducing the feature size within the porous polymers because of the scattering of light. Herein, although superhydrophobicity and transparency are generally two conflicting properties that are not easily improved simultaneously, they can be compromised through the optimal experiment.

**Figure 3:**
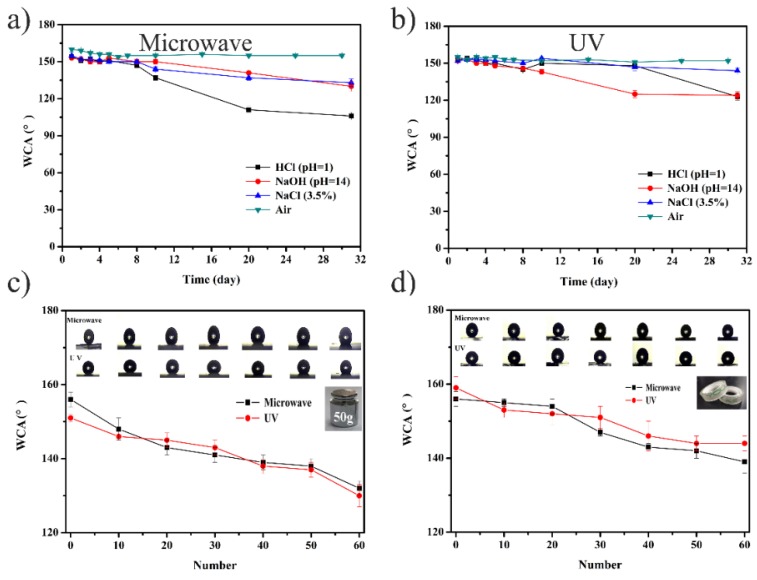
The change of WCAs for both monolithic coatings in air, acid, base and salt for 31 days (**a**,**b**), repeatedly abrasion (**c**) and peel-off (**d**) experiments for both monolithic coatings, respectively.

In order to examine the long-term preservation of superhydrophobicity, the glass plates coated with the monolithic coatings are kept in an open-air condition, different extreme environments for 31 days, respectively. As shown in Figure 3, no obvious change of the WCAs is found for both glass plates in the open-air condition, respectively and the surfaces still remain superhydrophobic in one month. The WCAs decrease gradually after both glass plates are immersed more than 10 days in aqueous solutions of pH = 1, pH = 14 and 3.5% NaCl but all monolithic coatings still remain hydrophobic after immersion in 31 days, respectively.

Durability of the coating is further tested against abrasion on the plastic desk with a load of 50 g, wherein the surfaces are abraded for a length of 10 cm, back and forth. Furthermore, the monolithic coatings are drawn repeatedly against a polyester wipe under a pressure of 10 kPa (using dead weight). For all the tests, the WCAs are measured after each 10 consecutive cycles and plotted in Figure 3c,d. Compared to those original states, the final decrease of WCAs is less than 15% after the abrasion and peel-off tests for 60 cycles, respectively.

In addition, the mechanical stability is also tested by bringing the as-prepared glass slides under a low velocity water jets and letting water drops impinge two typical monolithic coatings from 2 cm (0.6 m/s) and 10 cm (1.4 m/s), respectively (See Table S3 and Figure S7). For the coatings irradiated by microwave, no obvious decrease of WCAs is found after 35,100 and 27,000 drop impacts, with a falling height of 2 cm and 10 cm, respectively. The final WCAs are 152° and 142° accordingly. In contrast, a gradual decrease of WCAs is found for the coatings irradiated by UV with the final WCAs of 124° and 117° but still remain hydrophobic. From the results of chemical and mechanical properties, the ruggedness for both optimal polymers on the glass slides are better or close to some previous reports [24,25,26,27].

Likewise, other thin substrates were spread in two glass slides for the preparation of monolithic coating including the filter papers, aluminum coil, copper foil and poly tetra fluoroethylene (PTFE) film. Photos of water droplets of acid, base and salt on their surfaces are presented, respectively (see Figure S8). It should be noted these monolithic coatings are fabricated by UV irradiation. Metal foils must be inhibited for the irradiation in order to avoid the combustion due to the microwave interaction.

### 3.3. Application of the Prepared Filter Paper

**Figure 4:**
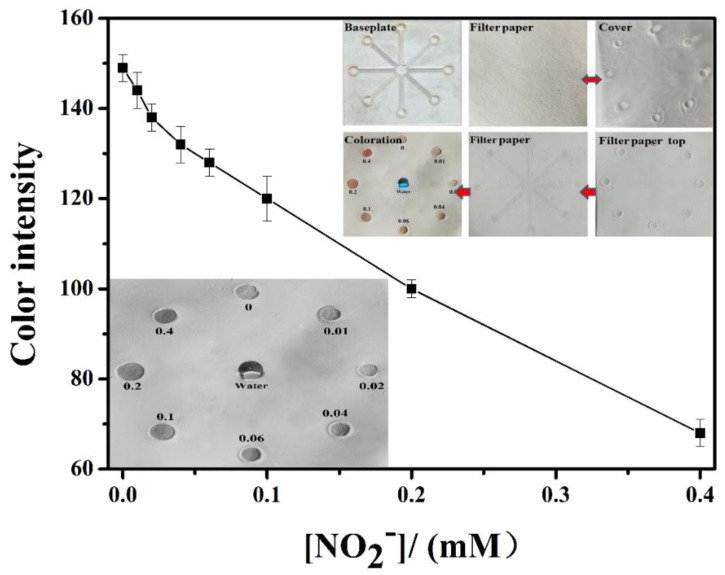
Colorimetric assays fabricated on the substrates of superhydrophobic filter papers. Nitrite assay based on the Griess reaction; the inset shows the prepared procedures (upside) and the analyte concentration in mM (downside), respectively.

One filter paper is attempted to be used as a microfluidic paper-based analytical device (μPADs) for the practical application [28,29]. The filter paper is firstly placed on a plastic support with 8 designed holes (holes of 4 mm in diameter) followed by dropping some polymerization solution of mixture 5, a UV-transparent cover plate with 8 opposite holes is then covered on the plastic support. After UV irradiation about 30 min, the filter paper modified by porous polymers with 8 hydrophilic dots is obtained but water droplets roll off easily on other regions of the whole filter paper except for 8 dots. It is attributed to that the solution dropped on the region with 8 holes leaked or evaporated without the support of the baseplate and effective shield of the upside cover. These hydrophilic dots can be used for introduction of standards and real sample for colorimetric detection. Griess reagent for the detection of nitrite is prepared with 50 mM sulfanilamide and 10 mM naphthalene ethylenediamine hydrochloride in methanol solution. Initially, 10 μL of this indicator solution is pipetted onto the reaction zones; following by 10 μL different concentrations of nitrite solutions are then added. A magenta azo compound is thus formed. The color results are analyzed by taking photos with a Huawei P10 smartphone and process with the Photoshop program (see Figure 4). These generated magenta stains become stronger upon increasing the nitrite concentration in the range of 0–0.4 mM. In general, RGB (red, green, blue) colors are the three major determinants. The images that are collected by the smartphone are digitized into RGB color coordinates and show non-monotonic behavior with light wavelength and intensity [30]. After these captured colors are transformed to the grey values using greyscale mode with the help of Photoshop software, data are imported into Origin (version 7.5) to obtain linear correlation between grey intensity (GI) and nitrate concentration (C). Herein, a linear correlation of GI = −194.6 C + 142.6 is obtained with a correlation coefficient of 0.9896 and a detection limit of 0.04 mM for the standard nitrite assay. These results are comparable with the data reported by the previous report [28,29,31], which is potentially used for the nitrate detection of real samples.

## 4. Conclusions

Two approaches of microwave and UV irradiation are comparatively attempted for the fabrication of monolithic coatings on the glass slide and filter paper conveniently. One step polymerization can be carried out in 10 min under an open-air condition for both methods. Both optimal polymers on the glass plates reveal distinct structures of porous network with long-term ruggedness in some extreme conditions. With the help of the microwave irradiation, the fabricated coatings on the glass slides exhibit larger WCAs but less transparency under the optimal conditions. It is believed the porogenic agents with less polarity are responsible for the better superhydrophobicity for the resulted monoliths. Most importantly, the superhydrophobic filter paper with some designed hydrophilic dots are readily patterned by UV photopolymerization and adapted for quantitative (nitrite detection) assays. Extended research with the developed method on different substrates for the creation of other more advanced μPADs are currently doing in our laboratory.

## Figures and Tables

**Table 1:** Preparation conditions for the monolithic coatings of poly(BMA-co-EDMA) and their water contact angles (WCAs) using ultraviolet (UV) irradiation.

Mixture No.	Monomer	Crosslinker	Porogen		Percentage of Porogen (%)	Average WCAs (°)
	BMA (μL)	EDMA (μL)	Butanediol (μL)	1-Propanol (μL)		UV
1	600	400	156	244	29	94°
2	600	400	233	367	38	100°
3	600	400	350	550	47	107°
4	600	400	506	794	57	95°
5	600	400	700	1100	64	109°
6	600	400	1167	1833	75	95°

## Supplementary Materials

The following are available online at https://www.mdpi.com/2079-4991/9/3/411/s1, Scheme S1: Fast and simple fabrication of superhydrophobic coating on the glass slide and filter paper by microwave and UV irradiation, Figure S1: The values of WCAs on the monolithic surface before and after CVD for mixtures 1-6 by UV irradiation, Figure S2: (**a**) FTIR and (**b**) TG measurements for the UV-cured monolithic coatings, Figure S3: Determination of XPS for the glass sliders with the help of microwave and UV irradiation. (**a**) XPS survey for the microwave-irradiated surface, (**b**) c 1s XPS spectrum, (**c**) O 1s XPS spectrum; (**d**) XPS survey for the UV-irradiated surface, (**e**) c 1s XPS spectrum, (**f**) O 1s XPS spectrum, Figure S4: Thickness of the fabricated monolithic coatings of mixture 5 on the glass sliders by the microwave and UV irradiation, Figure S5: Self-cleaning for the monolithic coating of mixture 5 irradiated by microwave, Figure S6: Drop bouncing on superhydrophobic surface for the monolithic coating of mixture 5 irradiated by microwave, Figure S7: Testing of the coating’s long-term resistance against drop impact, Figure S8: The states of water droplets of acid (pH = 1, orange), base (pH = 14, blue) and salt (3.5% NaCl, yellow) sitting on the (**a**) glass slider, (**b**) qualitative filter paper, (**c**) quantitative filter paper, (**d**) Cu foil, (**e**) Al foil, (**f**) PTFE film. The colors orange, blue and yellow are dyed with azorubin, rhodamine B and lemon yellow, respectively, Table S1: Preparation conditions for the monolithic coatings of poly (MAA-co-EDMA) and the WCAs using UV irradiation, Table S2: Preparation condition of the monolithic coatings fabricated using microwave and UV irradiation and their average WCAs, Table S3: The testing condition and WCAs for the durability of the coating towards long-term drop impact, Video S1 showed the self-cleaning on the superhydrophobic poly(BMA-co-EDMA) porous polymeric surface, Video S2 showed the water repellent property on the glass slider prepared by UV irradiation, Video S3 showed the behavior of water on two different gloves coated with the superhydrophobic poly (BMA-co-EDMA) powders.
