# Supplementary material for: Fast and Simple Fabrication of Superhydrophobic Coating by Polymer Induced Phase Separation
Source: Nanomaterials (Basel). 2019 Mar 11;9(3):411. doi: 10.3390/nano9030411 (PMC6473969; doi:10.3390/nano9030411)
Supplement: Supplementary file 1 [file nanomaterials-09-00411-s001.zip › nanomaterials-435424-SI.pdf]

Supporting Information:

Fast and Simple Fabrication of Superhydrophobic Coating by Polymer Induced Phase Separation

Yu-Ping Zhang [1,*], Pan-Pan Li [1], Peng-Fei Liu [1], Wan-Qing Zhang [1], Ji-Chao Wang [1], Cheng-Xing Cui [1,2], Xiang-Jun Li [3] and Ling-Bo Qu [2,*]

[1] Henan Institute of Science and Technology, Xinxiang 453000, China; ccxcyh@gmail.com (P.-P.L.); lpf8856@163.com (P.-F.L.); zhangwqzzu@163.com (W.-Q.Z.); wangjichao@hist.edu.cn (J.-C.W.); chengxingcui@hist.edu.cn (C.-X.C.)

[2] College of Chemistry and Molecular Engineering, Zhengzhou University, Henan 450001, China

[3] School of Chemical Sciences, University of Chinese Academy of Sciences, Beijing 100049, China; lixiangj@ucas.ac.cn

* Correspondence: zhangyuping@hist.edu.cn (Y.-P.Z.); qulingbo@zzu.edu.cn (L.-B.Q.);
Tel.: +86-373-304-0926 (Y.-P.Z.)

Table of Contents

I. Preparation of the superhydrophobic monolithic surfaces

II. Supplementary tables

III. Supplementary figures

Ⅳ. Legends for supplementary videos.

I. Preparation of the superhydrophobic monolithic surfaces

To prepare the superhydrophobic poly(MAA-co-EDMA) and poly(BMA-co-EDMA) coatings on different substrates, the polymerization mixture is dropped on their surfaces then cover a glass (quartz) plate followed by microwave or UV-initiated polymerization for 5 min and 30 min, respectively. After microwave and UV irradiation, respectively, the superhydrophobic coatings are finally obtained by opening the glass slide using a razor blade and washing with methanol.

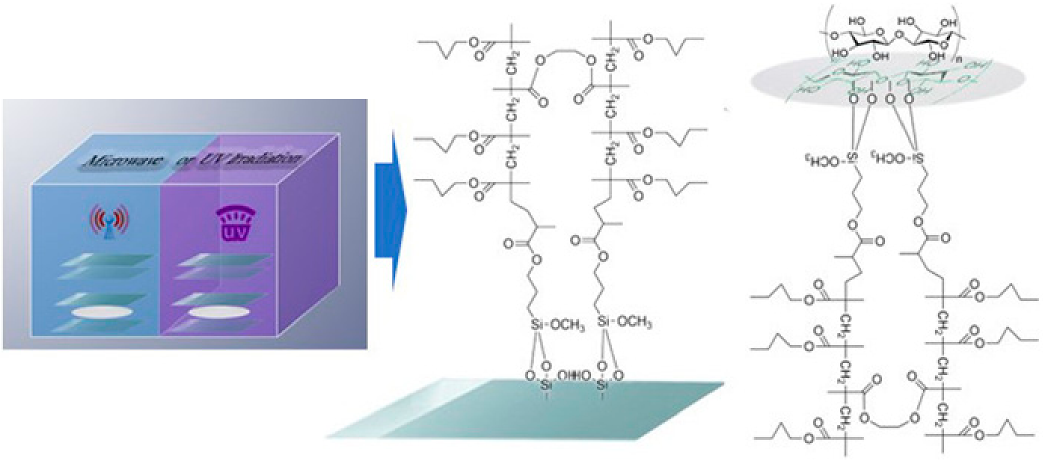

Scheme S1. Fast and simple fabrication of superhydrophobic coating on the glass slide and filter paper by microwave and UV irradiation.

II. Supplementary tables

Table S1. Preparation conditions for the monolithic coatings of poly (MAA-co-EDMA) and the WCAs using UV irradiation.

Mixture No.	Monomer	Crosslinker	Porogen		Percentage of porogen (%)	Average WCAs (°)
	MAA (μL)	EDMA (μL)	Toluene (μL)	Isooctane (μL)		UV
1	1560	13450	0	0	0	111
2	763	7635	6000	1500	50	85
3	600	5400	7200	1800	60	80
4	480	4100	8400	2100	70	77
5	300	2700	9600	2400	80	71
6	230	2050	10200	2520	85	100

Table S2. Preparation condition of the monolithic coatings fabricated using microwave and UV irradiation and their average WCAs.

Mixture No.	Monomer	Crosslinker	Porogen		Average WCAs (°)	
	BMA (μL)	EDMA (μL)	Butanediol (μL)	Lauryl alcohol (μL)	Microwave	UV
1	600	400	0	0	30	30
2	600	400	1500	0	152	146
3	600	400	1000	500	156	148
4	600	400	750	750	155	152
5	600	400	500	1000	170	155
6	600	400	0	1500	143	143

Table S3. The testing condition and WCAs for the durability of the coating towards long-term drop impact.

Method	Height (cm)	Time (h)	Velocity (m/s)	Droplet number (n)	Droplet radium (mm)	Average WCA (°)
Microwave	2	13	0.6	35100	3	152
	10	13	1.4	27000	3	142
UV	2	13	0.6	35100	3	124
	10	13	1.4	27000	3	117

III. Supplementary Figures

A precursor mixture consisting of monomers, porogenic mixtures, and a thermal/photo initiator is spread evenly on a substrate and then irradiation by microwave and UV, respectively. After ethanol flushing to remove the porogens from the substrate, microtextured superhydrophobic monolithic coatings are obtained. In the PIPS process,

the solvents are miscible with the cross-linkable monomers but immiscible with the generating network polymers, so that a phase-separated state can be obtained.

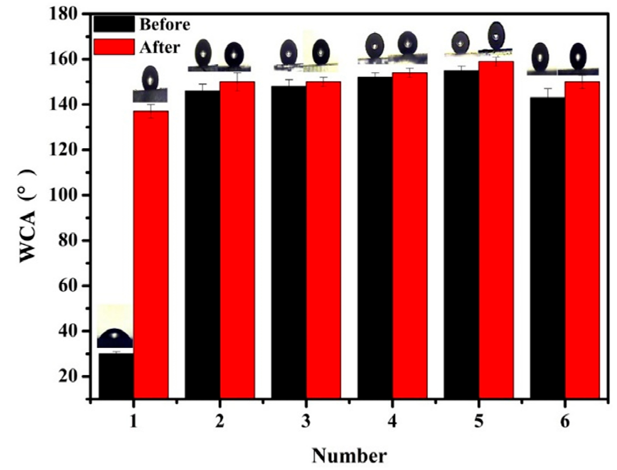

Figure S1. The values of WCAs on the monolithic surface before and after CVD for mixtures 1-6 by UV irradiation.

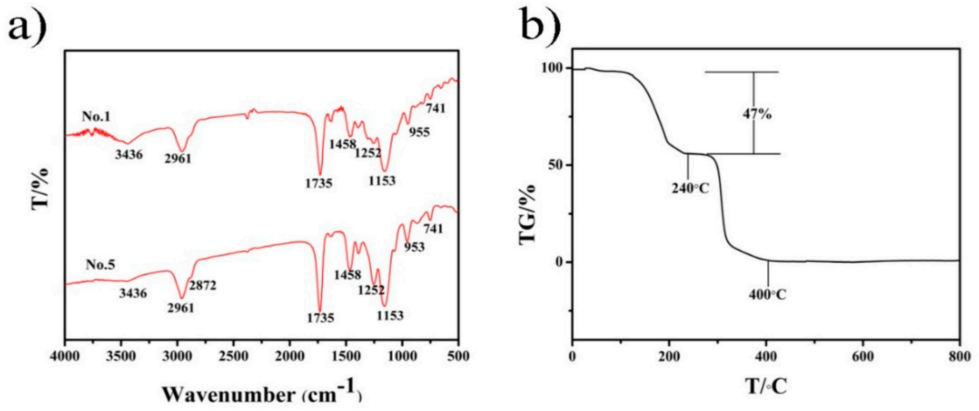

Figure S2. (**a**) FTIR and (**b**) TG measurements for the UV-cured monolithic coatings.

FT-IR are acquired using a 7600 infrared spectrometer (Lambda Scientific Pty.,Ltd) in Figure 2Sa. About 1-2 mg polymeric coatings of mixtures 1 and 5 are scraped down from the glass surface and mixed with 100 mg KBr, After the mixture is laminated evenly, then pressed into transparent sheet with 5 x10^7Pa pressure force. No obvious difference of spectra is found for mixtures 1 and 5. In general, the adsorption peak located at 2961 cm^{-1} is attributed to the stretching/vibration of-CH_3 and -CH_2, while the (C=) stretching/vibration of acrylate occurred located at 1735 cm^{-1}. The carbonyl (C=O) stretching band occurs at 1600 to 1850 cm^{-1}. The band at 1458 cm^{-1} is attributed to the skeleton vibration peak of benzene ring.

TG measurement is carried out and the results are shown in Figure 2Sb. 1 mL of solution (mixture 5) is injected into a UV transparent vial and irradiated by UV about 30 min after degassed with nitrogen about 3 min. The fabricated monoliths are flushed with ethanol for several times. After drying and fining, 10 mg of particles are used for the TG

determination. It indicates that there are 47% composition lost at a temperature of 240 °C and the rest lost at a temperature of 400 °C. The former is the unreacted monomer and porogens, and the latter is the cracked polymers of poly(BMA-co-EDMA).

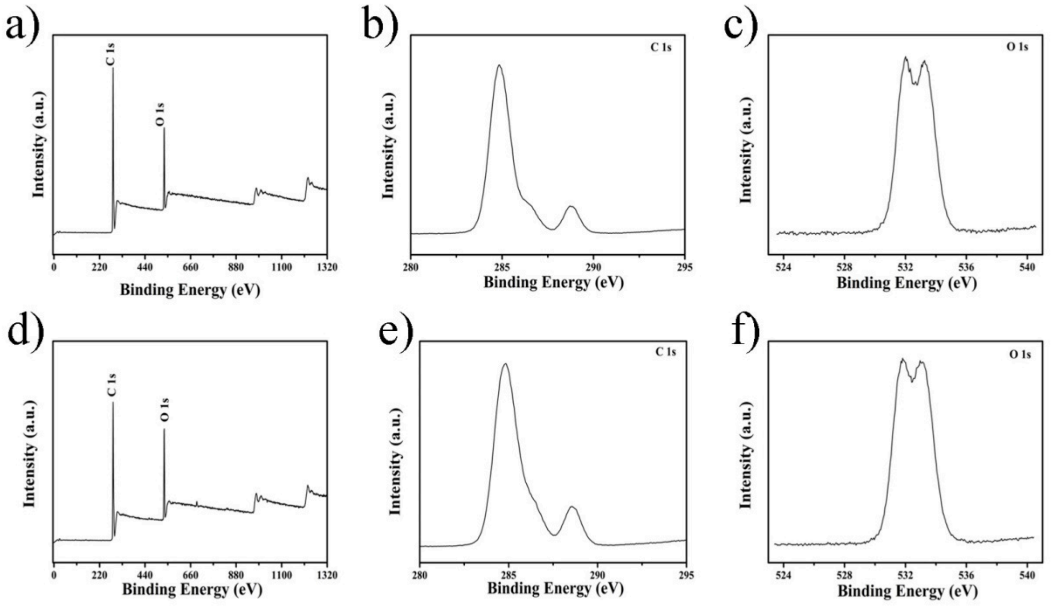

Figure S3. Determination of XPS for the glass sliders with the help of microwave and UV irradiation. (**a**) XPS survey for the microwave-irradiated surface, (**b**) c 1s XPS spectrum, (**c**) O 1s XPS spectrum; (**d**) XPS survey for the UV-irradiated surface, (**e**) c 1s XPS spectrum, (**f**) O 1s XPS spectrum.

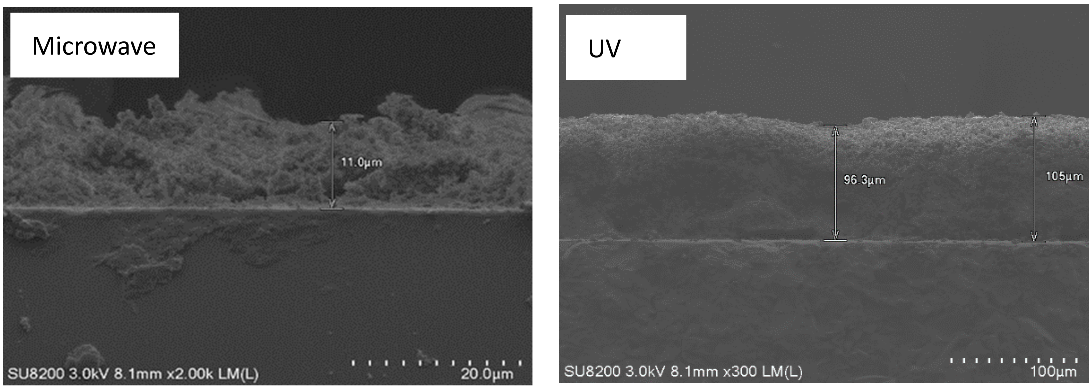

Figure S4. Thickness of the fabricated monolithic coatings of mixture 5 on the glass sliders by the microwave and UV irradiation.

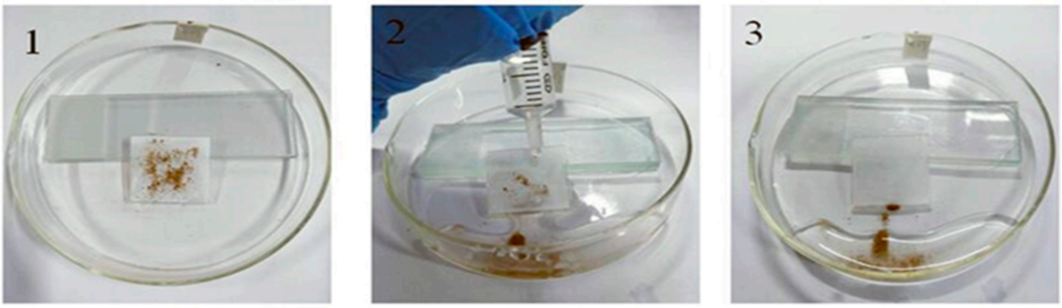

Figure S5. Self-cleaning for the monolithic coating of mixture 5 irradiated by microwave.

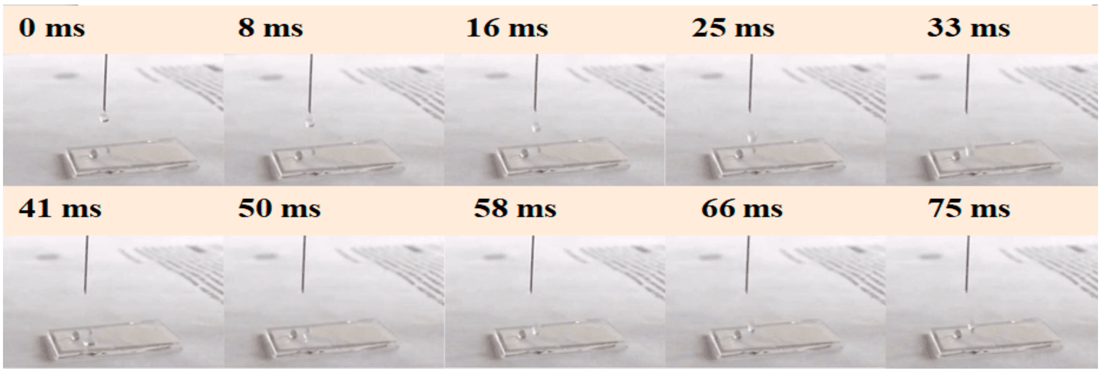

Figure S6. Drop bouncing on superhydrophobic surface for the monolithic coating of mixture 5 irradiated by microwave.

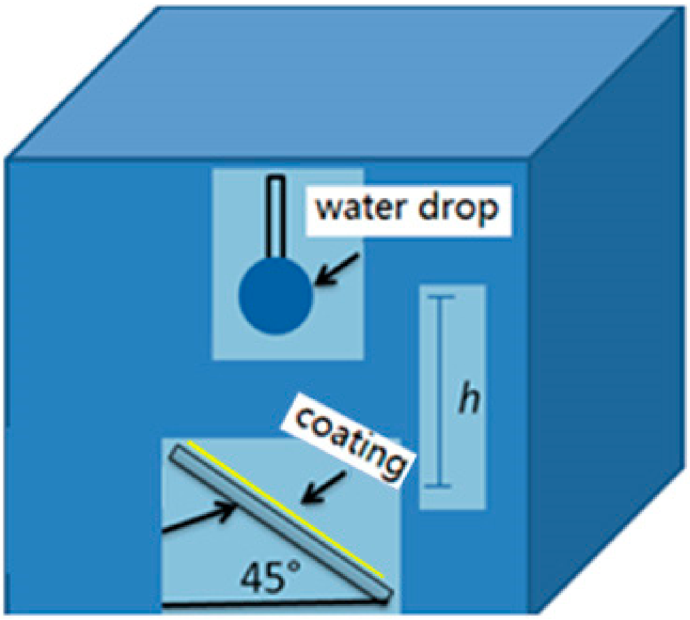

Figure S7. Testing of the coating's long-term resistance against drop impact.

Water drops about 3 mm size impinge two typical monolithic coatings from a falling height of 2 cm and 10 cm, respectively. The substrate is tilted by 45° and fixes above a sink to ensure water drain off. For the coatings irradiated by microwave, no obvious decrease of WCAs is found after 35,100 and 27,000 drop impacts, with a falling height of 2 cm and 10 cm, respectively. The final WCAs are 152 °and 142°, respectively. In contrast, a gradual decrease of WCAs is found for the coatings irradiated by UV with the final WCAs of 124 °and 117°, respectively, but still remain hydrophobic. The radium of water is determined according to the formula of $V = 4/3\pi r^3$.

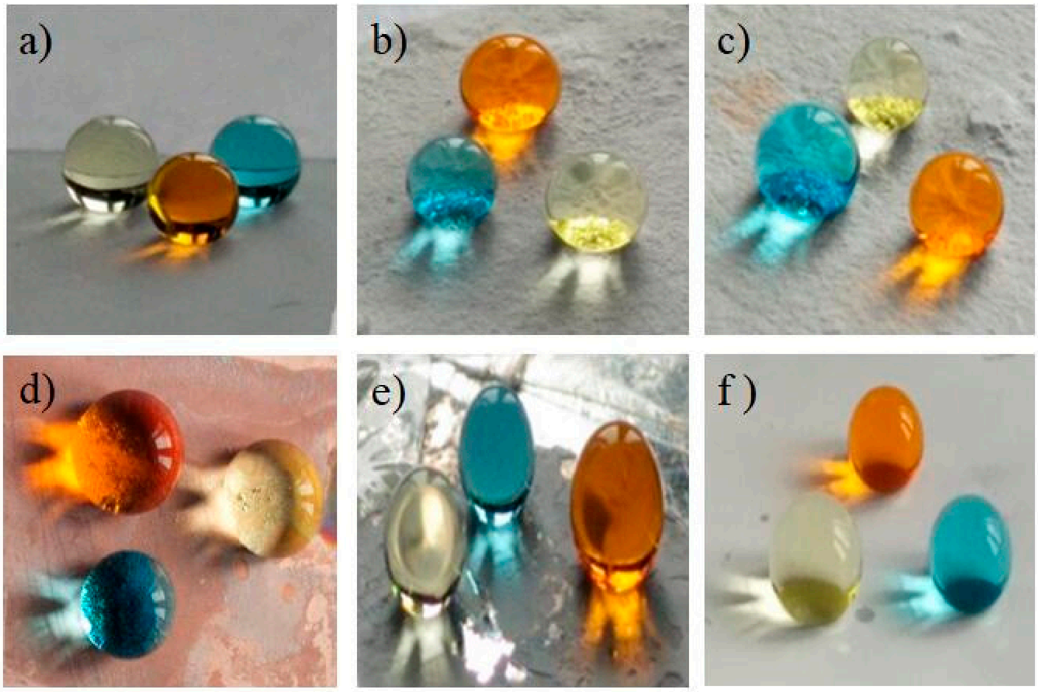

Figure S8. The states of water droplets of acid (pH = 1, orange), base (pH = 14, blue) and salt (3.5% NaCl, yellow) sitting on the (**a**) glass slider, (**b**) qualitative filter paper, (**c**) quantitative filter paper, (**d**) Cu foil, (**e**) Al foil, (**f**) PTFE film. The colors orange, blue and yellow are dyed with azorubin，rhodamine B and lemon yellow, respectively.

Ⅳ.Legends for supplementary videos.

Video S1 showed the self-cleaning on the superhydrophobic poly(BMA-co-EDMA) porous polymeric surface.

Video S2 showed the water repellent property on the glass slider prepared by UV irradiation

Video S3 showed the behavior of water on two different gloves coated with the superhydrophobic poly (BMA-co-EDMA) powders.
